# Purposive Facebook Recruitment Endows Cost-Effective Nutrition Education Program Evaluation

**DOI:** 10.2196/resprot.2713

**Published:** 2013-08-15

**Authors:** Barbara Lohse, Patricia Wamboldt

**Affiliations:** ^1^Department of Nutritional Sciences, The Pennsylvania State UniversityUniversity Park, PAUnited States

**Keywords:** nutrition education, folic acid, family meals, low-income, food security, Facebook, SNAP-Ed

## Abstract

**Background:**

Recent legislation established a requirement for nutrition education in federal assistance programs to be evidence-based. Recruitment of low-income persons to participate and evaluate nutrition education activities can be challenging and costly. Facebook has been shown to be a cost-effective strategy to recruit this target audience to a nutrition program.

**Objective:**

The purpose of our study was to examine Facebook as a strategy to recruit participants, especially Supplemental Nutrition Assistance Program Education (SNAP-Ed) eligible persons, to view and evaluate an online nutrition education program intended to be offered as having some evidence base for SNAP-Ed programming.

**Methods:**

English-speaking, low-income Pennsylvania residents, 18-55 years with key profile words (eg, Supplemental Nutrition Assistance Program, Food bank), responded to a Facebook ad inviting participation in either Eating Together as a Family is Worth It (WI) or Everyone Needs Folic Acid (FA). Participants completed an online survey on food-related behaviors, viewed a nutrition education program, and completed a program evaluation. Facebook set-up functions considered were costing action, daily spending cap, and population reach.

**Results:**

Respondents for both WI and FA evaluations were similar; the majority were white, <40 years, overweight or obese body mass index, and not eating competent. A total of 807 Facebook users clicked on the WI ad with 73 unique site visitors and 47 of them completing the program evaluation (ie, 47/807, 5.8% of clickers and 47/73, 64% of site visitors completed the evaluation). Cost per completed evaluation was US $25.48; cost per low-income completer was US $39.92. Results were similar for the FA evaluation; 795 Facebook users clicked on the ad with 110 unique site visitors, and 73 completing the evaluation (ie, 73/795, 9.2% of ad clickers and 73/110, 66% of site visitors completed the evaluation). Cost per valid completed survey with program evaluation was US $18.88; cost per low-income completer was US $27.53.

**Conclusions:**

With Facebook we successfully recruited low-income Pennsylvanians to online nutrition program evaluations. Benefits using Facebook as a recruitment strategy included real-time recruitment management with lower costs and more efficiency compared to previous data from traditional research recruitment strategies reported in the literature. Limitations prompted by repeated survey attempts need to be addressed to optimize this recruitment strategy.

## Introduction

A diet rich in nutrients and metabolites, as well as a physically active lifestyle with a balance between energy intake and expenditure are established components of health and vigor. Public health campaigns focus on nutrition education as a preventive medicine approach. Nutrition education has been defined as “…any combination of educational strategies accompanied by environmental supports, designed to facilitate voluntary adoption of food choices and other food- and nutrition-related behaviors conducive to health and well-being and delivered through multiple venues, involving activities at the individual, institutional, community, and policy levels” [1]. This definition has been adopted by the Supplemental Nutrition Assistance Program Education (SNAP-Ed), which is the educational arm of the Supplemental Nutrition Assistance Program (SNAP), formerly known as the food stamp program. SNAP-Ed, which is administered by the Food and Nutrition Services (FNS) of the United States Department of Agriculture, has budgeted US $401 million in Federal Fiscal Year 2014 to provide sound nutrition education to persons eligible to participate in SNAP and other income-based federal assistance programs [2].

Requirements for these nutrition education programs, as outlined in the SNAP-Ed Guidance [3], follow from the Healthy, Hunger-Free Kids Act of 2010 (Public Law 111-296), Section 241 that amends the Food and Nutrition Act of 2008. It includes, among other mandates, that SNAP-Ed activities are *evidence-based* [3]. Not as stringent as the Institute of Medicine or the National Institute of Health’s Roadmap, but aligned with the transdisciplinary model to accommodate behavioral and social sciences [4], an evidence-based approach in SNAP-Ed activities is defined as “…the integration of the best research evidence with the best available practice-based evidence. The best research evidence refers to relevant rigorous nutrition and public health nutrition research including systematically reviewed scientific evidence. Practice-based evidence refers to case studies, pilot studies, and evidence from the field on nutrition education interventions that demonstrate obesity prevention potential” [3]. FNS expects SNAP-Ed practitioners to offer interventions with an evidence base derived from either a review of research or a SNAP-Ed operator-led evaluation documenting that the intervention is meaningful to the intended audience and has a desired impact on behavior [3]. Across disciplines, a critical component of establishing the evidence base for an intervention or treatment is the client perspective [4]. Program evaluation documents the clients’ needs, values, and perspectives and thus, is an integral step in the process toward developing evidence-based practice.

Challenges in designing and implementing cost-effective and useful public health program evaluations have been well documented [5-9]. A related Cochrane Collection review recommended the need for more good-quality studies with interventions that effectively promote adherence to dietary advice [10]. Nutrition education programs that are not evaluated by the target audience do not fulfill any criteria of being evidence-based. Therefore, to implement the SNAP-Ed Guidance, attention to recruiting SNAP-eligible persons to nutrition education evaluation activities is vital.

Facebook was shown to be a cost-effective, useful tool to recruit young adults to a tobacco use survey [11] and the advertising mechanism of Facebook allowed effective and low cost recruitment of low-income women to a nutrition education program. However, retention to view and evaluate the nutrition program was not tested [12]. Review of literature on social media use supported studies of mechanisms to monitor and enhance health communication quality [13]. Thus, the purpose of our study was to examine Facebook advertising as a strategy to recruit participants, especially SNAP-eligible persons, to view and evaluate an online nutrition education program intended to be offered with some evidence base for SNAP-Ed programming.

## Methods

### Study Design and Recruitment

Facebook advertising was the recruitment strategy for two digitally delivered nutrition programs: *Eating Together as a Family Is Worth It* (*WI*) and *Everyone Needs Folic Acid* (*FA*). Facebook advertising offers two billing mechanisms: cost per click on the ad (CPC) or cost per appearance (or impression) of the ad to the target audience (CPM). Actual charges for each mechanism are based on competition for the target audience. A range of competitive bids is suggested at the time of ad development, which can be revised at any time to enhance ad competitiveness [14]. For each program evaluation, 3 routine Facebook advertising set-up functions were determined: costing action, daily spending cap, and population reach. Both the *WI* and *FA* evaluations utilized the CPC costing option; CPC bids were revised twice during the course of each evaluation to increase Facebook page impressions. Expenditures for each program were limited to US $100/day.

Audience reach (ie, the number of people who will see the ad) was calculated by Facebook, which was influenced by a number of demographic limitations (eg, age range, geolocation, and gender). Reach projections were revealed while delimiters and key words were entered. Ad development was considered complete when the projected target audience reached 124,460 and 201,380 for *WI* and *FA*, respectively. Ads targeted English-speaking, low-income Pennsylvania residents, 18-55 years with key profile words (eg, SNAP, food bank, and need money). *WI* and *FA* Facebook impressions each consisted of a short title, image with caption, and brief text, which included the ability to earn a US $15 gift card (Table 1). Finalized ads were submitted to Facebook for approval. The Institutional Review Board of the Office of Research Protections at Pennsylvania State University approved the studies and participants consented online. After clicking on the Facebook ad, respondents were directed to the welcome page on our secure website and left the Facebook platform. Confidentiality of the participants was maintained by unique codes for identification, securely encrypted data, and storage in password protected computers and servers.

**Table 1:** Facebook ads.

Facebook ad			Cost per campaign	Survey started	Program evaluation	Low-income^a^ survey started	Low-income^a^ program evaluation
**Eating Together as a Family is Worth It**							
	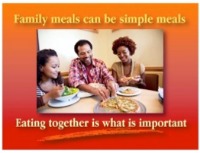	Let Penn State study know of your family meals and if our info helps. Earn US $15 Walmart Card	US $1197.45	US $16.40	US $25.48	US $32.36	US $39.92
**Folic Acid is for Everyone**							
	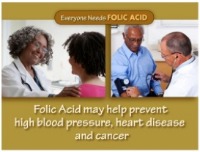	Earn US $15 gift card instantly for your thoughts on a Penn State research lesson: Folic Acid	US $1321.52	US $12.01	US $18.88	US $24.03	US $27.53

^a^Low-income defined as sometimes, often, or always worry about money for food and/or use of an income-based assistance program.

### Data Collection Process and Instruments

**Figure 1:**
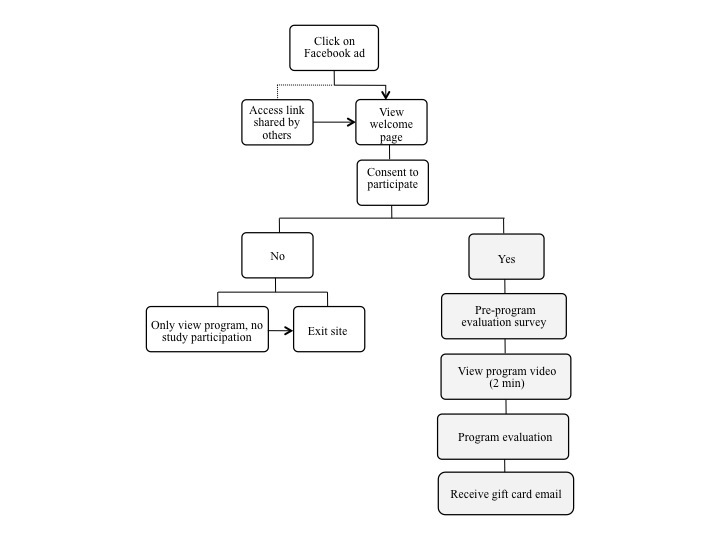
Program recruitment and evaluation path.

Data collection was staggered: *WI* data collection was completed prior to the start of the *FA* evaluation. Recruitment was realized with a click on the impression, which linked to the informed consent and agreement to participate (Figure 1). Participation required access to the survey link; the open survey format did not require password entry. As shown in Figure 1, respondents who clicked on the ad were linked directly to a Qualtrics platform (version 1527s, Qualtrics Labs Inc., Provo, UT; 2013), website that included a welcome page and a study consent form. On agreement to participate, subjects were invited to (1) complete a profile-oriented pre-evaluation survey, (2) view the program, and (3) respond to program evaluation survey items. In all, respondents completed a 53-item survey delivered across 13 screens.

The pre-program evaluation survey set identified demographics (9 items, included self-report height and weight), meal and food preparation habits (5 items), Internet and Facebook use (5 items), and nutrition assistance program use (eg, SNAP, food banks; 10 items). The survey set included the Satter Eating Competence Inventory for Low-Income (ecSI/LI), a 16-item, validated measure to assess eating competence (EC) [15]. EC refers to an intra-individual approach to eating and food-related behavior [16] that has been associated with several positive health outcomes, including less disordered eating [17], fewer cardiovascular risk factors [18,19], higher dietary quality [19,20], and greater physical activity [21]. ecSI/LI response options ranged from never or rarely (0 points) to always (3 points) so that the total score possible ranges from 0 to 48. Four subscales correlate with the 4 constructs of EC: Eating attitudes; eating context skills (eg, planning healthful meals); food acceptance; and internal regulation of intake. Possible subscale scores range from 0 to 15 for eating attitudes and contextual skills and from 0 to 9 for the remaining subscales.

Traditionally, *FA* and *WI* were designed for delivery on a digital photo frame, video or computer monitor, or a power point show as education to view while waiting for services (eg, in a clinic, grocery store, or government agency). For purposes of this evaluation, each program was converted to a short video loop (approximately 2 minutes long), then inserted into the Web-based survey for viewing after completion of the pre-evaluation survey. A video loop eliminated the need for slide advancement, enhancing program viewing. Respondents were required to view the video at least once in its entirety before advancing to the program evaluation survey; however, the video loop could be viewed as many times as preferred prior to program evaluation.

The evaluation survey completed after viewing the program featured a variety of question types and response options. A list of program descriptors (8 items) was included and respondents selected all statements that were true for them. (eg, I learned a lot. This program was helpful.) Additionally respondents rated the amount of information in the program (not enough, the right amount, or too much) and the speed of the program (needed more time, had enough time, or moved too slowly). Individual slides were displayed and respondents rated each for likability, importance, or clarity with the Likert scale, heat map, and rating scale formats. Participants were encouraged to type in comments and suggestions.

### Data Analysis

Data were captured with Qualtrics and analyzed using the Statistical Package for Social Scientists (v 20, IBM SPSS, Armonk, NY; 2010). Facebook log data were compiled from Facebook ad manager campaign reports that included daily information such as number of impressions, click-through rates, and expenditure.

A 2-step process eliminated data from repeated survey attempts. First, computer Internet Protocol (IP) addresses were screened for frequency. If an IP address was duplicated, only the initial survey response, as determined by the survey time stamp, was included in the data set. After eliminating duplicate IP responses, email addresses with redundancy were assessed in the log file analyses. If email addresses were very similar, and demographic information (including age, number of children, height, and weight) matched, and the IP address indicated the same geographic area, then only the first survey was included in the data set.

*FA* evaluation also utilized Qualtrics’ *ballot-box stuffing* survey protection option as a measure to control repeated survey attempts by preventing a user from taking the survey from the same computer and browser. Participants completing the initial submission of IP-identical surveys with dissimilar entries for age, height, weight, number of children, ages of children, and email addresses were contacted by email to ascertain the probability that subsequent surveys from the same IP address were respondent duplicates. Email contact was made with the initial responder only. Responses to the email query that fit the data were retained. For example, we retained both surveys from the same IP address when our queries confirmed that one respondent was the mother and the other the daughter who used the same computer. Responses that did not fit or were questionable were not included.

Assistance program participation was identified by affirmation. Low-income was defined as using at least one of the means-based assistance programs *or* sometimes, often, or always worrying about money for food. ecSI/LI item responses were summed to provide total and subscale scores. EC was defined as a total score ≥32 [19]. Response rates were calculated as directed in the Checklist for reporting Results of Internet E-Surveys (CHERRIES) [22] using a unique IP address as a proxy for a unique site visitor. The informed consent page was denoted as the first page of the survey. The *view rate* was calculated as the ratio of unique visitors to the first survey page divided by the unique visitors to the study site (ie, the welcome page that appeared after clicking the Facebook ad). The *participation rate* was the number of study consenters divided by unique visitors to the informed consent page. Finally, dividing the number of respondents who completed the last survey page by the number who had agreed to participate was the *completion rate* [22].

## Results

### Participant Description

Characteristics of *WI* and *FA* program evaluators were similar (Table 2). In general, they were white, under 40 years, overweight or obese and dissatisfied with their weight (mean self-report body mass index, BMI: *WI*, mean 30.0, SD 7.4; *FA*, mean 29.7, SD 8.9), and not eating competent. The *WI* mean ecSI/LI score was: mean 29.76, SD 6.47 (range 12-41). Subscale means were: mean 9.96, SD 2.43 (eating attitude); mean 4.61, SD 2.30 (food acceptance); mean 6.02, SD 1.79 (internal regulation); and mean 8.94, SD 3.01 (eating context). The *FA* mean ecSI/LI score was: mean 28.71, SD 8.22 (range 12-48). Subscale means were: mean 10.19, SD 3.09 (eating attitudes); mean 4.66, SD 2.21 (food acceptance); mean 5.81, SD 2.16 (internal regulation); and mean 8.05, SD 3.26 (eating context).

For both studies, more than half were identified as low-income (*WI*: 37/59, 63%; *FA*: 55/77, 71%). An income-based assistance program was used by 39% (23/59) *WI* and 42% (32/77) *FA.* Always, often, or sometimes worrying about money for food was reported by 51% (30/59) *WI* and 60% (46/77) *FA.*

*WI* and *FA* respondents liked to cook or thought cooking was okay (48/57, 84%; 63/74, 85%, respectively). Most prepared meals at home at least 4 times a week (*WI*: 51/59, 87%; *FA*: 59/77, 77%) and spent 15-45 minutes preparing the meal (*WI*: 48/57, 84%; *FA*: 50/72, 69%). From a list of 5 meal preparation options, in which any or all could be selected, most chose home-style, made from scratch meals (*WI*: 50/72, 69%; *FA*: 51/77, 66%) with speed-scratch from mixes or meal kits ranking as the second most common (*WI*: 36/73, 49%; *FA*: 47/77, 61%). Meals described as healthy, low fat, low-sodium were selected less frequently (*WI*: 28/73, 38%; *FA*: 31/77, 40%).

Facebook access frequency was reported at least daily by 88% (53/60) *WI* respondents, and by 95% (73/77) *FA* respondents. Facebook recruitment revealed two paths: (1) clicking on the Facebook page impression; and (2) Web-link sharing (eg, in an email) by a Facebook friend (30/59, 51% for the *WI* study, and 22/77, 29% for the *FA* study).

### Recruitment and Response Rates

Facebook posted 4,278,732 and 4,192,197 impressions of the *WI* and *FA* ad, respectively, during the study timeline; the higher the daily/competing bid the more often the ad was displayed. *WI* responses were collected over 14 calendar days from April 24 to May 12, but paused on April 25, May 8-9, and part of May 10, for a total of 343 hours. The Facebook campaign was closed on May 15 at noon; however, the survey remained open to responses in Qualtrics for an additional 24 hours. Three surveys were submitted after the Facebook closing date and reached the study site by the second access pathway described above.

A total of 807 Facebook users clicked on the *WI* ad (which represented 807/124,460, 0.6% of potential reach) with 73 unique site visitors and 47 of them completing the program evaluation (ie, 47/807, 5.8% of clickers and 47/73, 64% of site visitors completed the evaluation). Completion pattern analyses revealed afternoon and evening as the most common times with 30% initiating the survey between noon and 6 pm and 47% after 6 pm. Average time spent on the survey site was 14 minutes.

*FA* data were collected over 17 calendar days from September 12 to 29, pausing on September 28 for a total of 384.3 hours reaching 0.4% (795/201,380) of potential accounts.

A total of 795 Facebook users clicked on the ad with 110 unique site visitors, and 73 completing the evaluation (ie, 73/795, 9.2% of ad clickers and 73/110, 66% of site visitors completed the evaluation). Average time spent on the survey site was 20 minutes. Similar to the *WI* program, 49% initiated the survey between noon and 6 pm and 38% after 6 pm.

IP address and log analyses identified 38 *WI* and 9 *FA* duplicate attempts at survey completion; these attempts were from 20 *WI* and 6 *FA* respondents. Psychosocial and demographic (eg, ecSI/LI score, age, self-report BMI, number of children, assistance program use, and low-income status) characteristics were similar between respondents making repeated attempts to complete the survey and those accessing the survey only once. Facebook campaign reach figures are displayed in Table 3.

For the *WI* program, CHERRIES view, participation, and completions rates were 88%, 97% and 76%, respectively. For *FA*, CHERRIES view, participation, and completions rates were 71%, 100%, and 95%, respectively.

Facebook recruitment campaign costs are summarized in Table 1. Evaluation costs related to respondent incentives and personnel are not included.

**Table 2:** Demographic characteristics of evaluators recruited using Facebook.^a^

		Full sample, n		PA residents, n		Low income,^b^ n	
		WI (n=59)	FA (n=77)	WI (n=27)	FA (n=73)	WI (n=37)	FA (n=55)
**Body mass index (BMI)** ^c^							
	Below 18.5 (underweight)	2	1	0	1	3	0
	18.5-24.9 (normal)	20	34	22	34	24	33
	25-29.9 (overweight)	39	20	33	21	24	22
	30 and above (obese)	34	42	41	40	41	42
**Eating competence** ^d^							
	Not eating competent	60	73	61	66	83	69
**Assistance program use** ^e^							
	Supplemental Nutrition Assistance Program	22	29	19	26	35	40
	Women, Infants, and Children (WIC)	24	21	22	19	38	29
	Cash assistance benefits	2	9	4	7	3	13
	Temporary assistance for needy families	2	7	4	4	3	9
	Medical assistance benefits	24	23	33	22	38	33
	Medicaid	14	12	19	8	22	16
	Medicare part D-prescription drug coverage	7	8	11	8	11	11
	Low income home energy assistance program	7	17	15	15	11	24
	Expanded food and nutrition education program	2	3	4	1	3	4
	Food bank or food pantry	15	12	26	10	24	16
**Education**							
	Less than high school	2	0	4	0	3	0
	High school graduate or equivalent	18	21	19	22	23	26
	Some college or 2-year degree	28	51	37	49	31	49
	4-year college degree	30	22	19	22	23	22
	Postgraduate college	23	7	22	7	20	4
**Number of children per household**							
	1 child	36	41	40	43	32	46
	2 children	44	32	33	33	41	35
	3 or more children	18	25	27	20	27	14

^a^Table entry is %, numbers may not add to100 because of rounding.

^b^Low income defined as sometimes, often, or always worry about money for food and/or government assistance program use.

^c^Self-reported height and weight were missing so BMI was not calculated for 5% of WI and 4% of FA participants.

^c^EC is defined as ecSI/LI score ≥32.

^e^More than one choice could be selected.

**Table 3:** Facebook campaign response (N=).

Campaign metric	Eating As a Family is Worth It	Everyone Needs Folic Acid
Clicks on Facebook ad	807	795
Clicks on study welcome page	111	119
Duplicate attempts removed from data set	38	9
Unique site visitors clicked on study welcome page from a unique IP address	73	110
Unique survey visitors visited informed consent page	64	77
Agreed to participate	62	77
Program evaluation started (ie, answered at least 1 item)	60	73
Program evaluation completed	47	73

## Discussion

### Principal Findings

This study demonstrated that Facebook was an efficacious and cost-effective strategy to recruit low-income persons to evaluate online nutrition education programs. Findings contributed to the growing body of evidence that Facebook is a useful, lower-cost tool for health communication [13,23], public health surveillance [24], recruitment to health-related surveys [25-27], and online interventions including those targeting weight [28] and physical activity [29].

### Cost Effectiveness and Campaign Management

The findings confirmed that Facebook-driven nutrition education recruitment efforts are a cost-effective means to reach low-income persons. Our costs of US $24.03 (*FA*) and US $32.26 (WI) to recruit each low-income person to a nutrition behavior survey were higher than the US $15.30 reported in an earlier study of Facebook as a strategy to recruit low-income persons [12]. That study, which attracted more participants, had a lower respondent burden because it focused only on recruitment and did not include a program evaluation component. Costs to recruit each low-income participant were much lower than the US $51.59 expended when traditional methods of flyers, postcards, and telephone calls were used [30]. Likewise, costs for each program evaluation completion were more than 55% (*WI*) to 70% (*FA*) lower than the US $94.36 incurred by these traditional recruitment methods [30]. The findings were not delimited by program message because one was nutrient-based and the other focused on family mealtime issues.

Disappointing Facebook recruitment efforts for pre- and perimenopausal women [31] and for targets audiences with conditions or characteristics not easily captured by key words (eg, depression) [32] have been reported. Therefore, like Chu and Snider [25] we adjusted CPC bids and campaign hours to capitalize on our target audience’s Facebook routines to promote reach.

### Reaching Low-Income Persons

Nearly two-thirds of respondents were low-income. Each Facebook campaign recruited a low-income sample similar in age, BMI, EC (overall and component constructs), level of worry about money for food [12,15,21,33,34], and food preparation habits [12] to those recruited for other nutrition education studies. Program evaluation was not superficial: 44% (21/48) of *WI* and 15% (11/73) of *FA* evaluators viewed the program more than once, completion rates were high (*WI*: 47/62, 76%; *FA*: 73/77, 95%), and useful program improvement comments were provided. One study limitation was the need to rely on a proxy definition of low-income (ie, worry about money for food or self-reported assistance program use) rather than confirmed records of income, which are difficult to secure and interpret. However, in addition to prior support for use of this index [12], its merit was supported because frequency levels of overweight and obesity (*WI*, *P*<.001; *FA*, *P*=.05) and frequency of a high school or less education (*WI*, *P*=.049; *FA*, *P*=.11) were greater for index-denoted low-income persons; similar to earlier reports [17], a smaller proportion of low-income persons were categorized as eating competent (*WI*, *P*<.001; *FA*, *P*=.05).

### Cautions: Chain Sampling and Repeat Survey Attempts

Facebook is a utility to encourage communication and sharing among friends and this extends to letting friends know how to access a survey with a gift card incentive. This “word of click” access (also known as “chain sampling” [27]) was especially noted in the *WI* evaluation. Although a means to enrich a dataset without paying for Facebook clicks, circumvention of eligibility criteria has the potential to contaminate study outcomes. Our goal to recruit Pennsylvania residents was a funder-imposed goal, thus we included non-Pennsylvania residents in our program evaluation findings and relied on Qualtrics’ skip logic to enforce nongeographic eligibility criteria. *WI* respondents living in other states were not dissimilar from the Pennsylvania residents in proportion of low-income, EC, education level, Internet use, amount of worry about money for food, BMI, liking to cook, or participation in SNAP or the Special Supplemental Nutrition Program for Women, Infants, and Children (WIC). The only difference noted was that 56% (15/27) of Pennsylvania residents reported cooking each day compared to 25% (8/32) of non-Pennsylvanians (*P*=.03). Only 4 *FA* respondents were not Pennsylvania residents; however, those 4 were similar to Pennsylvanians on all parameters measured with one exception—all out-of-state responders were EC. Consideration of “word of click” recruitment implications will depend on study goals and restrictions. For this study, the recruitment of a broader audience to evaluate the nutrition education programs, thereby improving the generalizability of our findings, was a plus. Fenner et al [27] also reported that the participants recruited from Facebook friends’ sharing the survey link (ie, chain sampling/word of click) did not influence study outcomes.

Providing gift cards, money, or prizes meant to incentivize study participation or reimbursing respondents for cost incurred to participate (eg, parking, childcare) and access to the survey site resulted in repeated attempts to access and complete the survey. The “ballot box stuffing prevention” feature of the Qualtrics platform prevents duplicate IP addresses from accessing the survey. However, using applications to ensure anonymity may nullify this protection. Our protocol of checking IP and email addresses with follow-up data review and telephone confirmation prevented duplicate payment and ensured database fidelity with minimal personnel effort.

### Future Research

Our focus was the evaluation of an online nutrition education program; however, numerous nutrition education programs (eg, WIC, Expanded Food and Nutrition Education Program) are based on face-to-face classes and meetings. Limited research is available to discern efficacy of Facebook to recruit persons to these on-site programs. Facebook campaigns successfully recruited young women to complete a health-related survey at a conveniently located study site and provided an option for completion online by those declining a site visit. An equal number agreed to come to the study site or accepted an invitation to complete the survey online [27]. The findings encourage use of Facebook to recruit to face-to-face nutrition education programs, especially if they are conveniently located. However, those opting for the online survey format were overweight and in the lowest socioeconomic status, thereby challenging recruitment to programs that serve low-income women, such as WIC.

Facebook ads specifically targeting low-income, Spanish-speaking Latinos could be tested for ability to recruitment to nutrition education programs. An online smoking cessation campaign was shown to be an effective way to recruit Spanish-speaking Latino smokers. However, this campaign utilized 4 websites that did not include Facebook and costs were US $209.34 per participant [35].

### Conclusion

This study confirmed that Facebook is a cost-effective strategy to recruit low-income persons to a nutrition program. Two separate evaluations of a nutrition education program supported the use of Facebook as a cost-effective strategy to additionally recruit low-income persons to view a nutrition education program and provide substantial evaluation. Potential application to recruitment for on-site nutrition education programs (eg, WIC) should be explored. Researchers are cautioned to monitor for duplicate survey attempts and to identify mandatory eligibility criteria to facilitate data management and analyses congruent with study aims.

## Abbreviations

BMI: body mass index
CHERRIES: Checklist for Reporting Results of Internet E-Surveys
EC: eating competence
ecSI/LI: Satter Eating Competence Inventory for Low-Income
FA: Everyone Needs Folic Acid
FNS: Food and Nutrition Services
IP: Internet Protocol
SNAP: Supplemental Nutrition Assistance Program
SNAP-Ed: Supplemental Nutrition Assistance Program Education
WI: Eating Together as a Family is Worth It
WIC: Special Supplemental Program for Women, Infants, and Children
